# Retrieval from the Brain's Perspective

**DOI:** 10.3389/fnhum.2012.00231

**Published:** 2012-08-06

**Authors:** Nikolai Axmacher, Juergen Fell

**Affiliations:** ^1^Cortical Oscillation Lab, Department of Epileptology, University of BonnBonn, Germany

Commonly used categories or axes to characterize retrieval processes like explicit (declarative) vs. implicit (non-declarative) memory or recollection vs. familiarity are intuitively plausible (Tulving, 1985). Everyone can distinguish between the phenomenal experience of “knowing” a person and of “remembering” a more detailed context of previous encounters with this person. Thus, these categories are without doubt relevant for an understanding of memory processes from a psychological perspective. However, it is still an open question whether they are actually optimal in a cognitive neuroscience framework – in other words, whether these categories reflect distinct operations within the brain, or whether they are rather epiphenomenal. Which criteria should neurocognitive categories characterizing memory retrieval (and encoding) fulfill? First, it should be possible to unequivocally operationalize these categories in experimental paradigms. Second, such categories should allow one to classify memory operations according to the involved brain structures and neural processes.

The explanatory value of the recollection vs. familiarity dichotomy is hampered by the difficulty to operationalize it appropriately. This issue has been highlighted by a recent review article (Voss et al., 2012) and a spotlight article (Paller et al., 2012). In these two papers, the authors have argued that implicit memory can drive behavioral responses on explicit memory tests aiming to measure recollection and familiarity, which may cause distorted results. More specifically, apparent familiarity signals may in many cases be actually due to conceptual priming. This corrupts the interpretation of activation patterns during familiarity judgments and renders reverse inference problematic (Poldrack, 2006). Other researchers have pointed to the fact that a separation between brain structures supporting recollection and familiarity is often confounded by memory strength (e.g., Wixted and Squire, 2010). It may be argued that, for instance, the categories of item vs. source memory can be more clearly operationalized and may better distinguish between involved brain structures and neural processes (e.g., Staresina and Davachi, 2008). While the assessment of recollection and familiarity typically relies on introspective reports that are inherently subjective, source memory can be objectively assessed.

Recently, Henke (2010) moved beyond this methodological point and argued that in principle, memory operations should be characterized by different processing modes, and not by the involvement of consciousness (explicit vs. implicit) or other phenomenal criteria. This line of reasoning was strongly motivated by neuroimaging studies suggesting that the criterion for the recruitment of a specific brain structure is related to its computational role rather than to the phenomenal experiences during the memory task. According to her new taxonomy, the hippocampus, for instance, accomplishes rapid encoding of flexible associations, while the parahippocampal gyrus supports rapid encoding of single items. Compared to the explicit vs. implicit dichotomy, the advantage of this classification is that its relation to different brain structures is less ambiguous.

In a similar vein, Ranganath (2010) recently concluded in a review article that the functional differences between medial temporal subregions do not precisely correspond to different types of memory tasks, cognitive processes, or states of awareness. In his opinion, present evidence indicates that medial temporal subregions mainly differ in terms of the kind of information they process and represent.

How could retrieval be investigated in a more mechanistic neuroscientific framework which is closely related to the putative operational characteristics of specific brain regions? One attempt to study retrieval processes using neurocomputational categories was based on the idea that source memory should be related to pattern completion, which might be implemented in specific hippocampal subregions (e.g., Norman and O’Reilly, 2003). More recently, the increasing application of multivariate pattern classification analyses (MVPA) to memory research enabled a detection of distributed patterns of category- or even stimulus-specific activity (Rissman and Wagner, 2012). This method thus allowed one to test the long-standing hypothesis that retrieval consists in a re-instantiation of stimulus representations (Tulving and Thompson, 1973). For example, MVPA of fMRI data revealed reoccurrence of category-specific activations initiated by retrieval, immediately before an item of this category was recalled (Polyn et al., 2005), and single-unit recordings in epilepsy patients even demonstrated reactivation of stimulus-specific cellular activity preceding memory retrieval (Gelbard-Sagiv et al., 2008). Similarly, a replay of spatially selective sequences of hippocampal place cells has been observed during memory retrieval in rats (Pastalkova et al., 2008). Importantly, reinstatement of BOLD activity patterns was also found during recognition memory, regardless of whether it was associated with familiarity or recollection judgments, although with a larger magnitude for recollection than familiarity responses (Johnson et al., 2009). Further studies are necessary to corroborate these findings. For the present, these results suggest that the investigation of retrieval processes by a mechanistic neurocognitive approach may result in different classifications of retrieval processes – with possibly fewer and better defined distinctions – than classifications based on phenomenally motivated categories.

To summarize, although there is a long tradition to use categories like explicit vs. implicit or recollection vs. familiarity in memory research, it is still an open question whether the brain actually operates with these categories. In our opinion it is an important challenge to explore whether there are other memory categories, like processing modes (Henke, 2010), item vs. source memory (Staresina and Davachi, 2008), or the degree of pattern instantiation (Johnson et al., 2009), which are more closely related to brain structures and neural processes, and to clarify how these categories relate to those commonly used.
